# Spatiotemporal Patterns of Impervious Surface Area and Water Quality Response in the Fuxian Lake Watershed

**DOI:** 10.1155/2020/4749765

**Published:** 2020-04-25

**Authors:** S. H. Li, L. Hong, B. X. Jin, J. S. Zhou, S. Y. Peng

**Affiliations:** ^1^Yunnan Provincial Geomatics Centre, Kunming, Yunnan, China; ^2^College of Tourism & Geographic Sciences, Yunnan Normal University, Kunming, Yunnan, China; ^3^Information Center, Department of Natural Resources of Yunnan Province, Kunming, Yunnan, China

## Abstract

The increase of urbanization level has led to the rapid increase of impervious surface area (ISA). The aim of this work is to clarify the relationship between the ISA and water quality and lay a foundation for the improvement and protection of the water quality in the basin. Taking the Fuxian Lake Basin in Yunnan Province as an example, based on the Landsat ETM+ remote sensing image and the Gram–Schmidt (GS) image fusion algorithm, the four-terminal model and the linear spectral mixture model (LSMM) were used to extract the impervious surface of the watershed from 2006 to 2015. And statistical methods were used to distinguish its relationship with water quality. The results show that the four-terminal model and the linear spectral mixture model can effectively extract the impervious surface information of the Fuxian Lake Basin. The average root mean square error (RMS) of the image decomposition results from 2006 to 2015 was less than 0.02. In the past 10 years, the ISA has changed significantly in the Fuxian Lake Basin. The ISA showed an overall upward trend from 2006 to 2015. It increased from 24.73 km^2^ in 2006 to 35.14 km^2^ in 2015, an increase of 10.81 km^2^. From the value anomaly, the ISA in 2006 and 2009 is lower than the multiyear average, and those in the other years are higher than the multiyear average. The percentage of ISA in the basin was significantly positively correlated with Chemical Oxygen Demand-Mn (CODMn) and total phosphorus (TP) (*r* is 0.772, 0.763), and the correlation in the flooding season was greater than that in the dry season. The ISA threshold for water quality deterioration is around 10% in the Fuxian Lake Basin. Reducing ISA coverage, controlling ISA to less than 10%, and preventing nonpoint source pollution during flooding season will be the best measures to effectively improve the water quality environment in the basin.

## 1. Introduction

A large number of pollutants including organic matter, suspended solids, heavy metals, pathogens, oils, and other toxic and hazardous substances are accumulated on the surface of urban roads, roofs, construction sites, etc. These pollutants are merged into water bodies such as rivers, lakes, and bays through impervious surfaces or drainage networks under the effect of rainfall erosion. Thus, it poses a serious threat to the water environment. Rainfall runoff and land covering represented by impervious surface area (ISA) are important factors in the formation of nonpoint source pollution. The relationship between the ISA and the water quality has always been the focus of regional ecological environmental effects. The effect of ISA on water quality is mainly due to the increase of nonpoint source pollution load of water bodies [1]. Most studies have found that the higher the ISA, the worse the water quality [2, 3]. The ISA coverage can predict the change of water quality chemical indicators to a certain extent. When the ISA coverage is less than 10%, the river water quality is relatively good [4]. The pH value in water is an important indicator for analyzing water quality. There is a strong correlation between pH value and ISA in water. The potential threshold range between the two is 2.4%–5.1% [5]. The ratio of the ISA of the subbasin has a significant linear relationship with the COD and NH3-N concentrations in the river [6]. Marinoni established a model for evaluating the impact of simulated land development on water quality indicators such as TN, TP, and TSS based on mathematical programming and combined with multiscale analysis and motion wave equations [7].

With the diversification of remote sensing data sources and the advancement of remote sensing technology, multisource remote sensing data provides the basis for multiscale effect analysis of ISAs. Among them, most people currently use medium- and high-resolution image fusion methods to extract ISA. It is not the data itself, but an alternative data source to satellite imagery that improves ISA mapping. Auxiliary data in matrix (raster) format may be useful to extract ISA. For example, the application of building density survey data, US national land use/cover classification data (NLSD), GPS field survey, land use data, and other vector data combined with low-resolution remote sensing data to estimate the distribution characteristics of ISA [8, 9]. The integration of multisource data with multiple technical methods is currently the mainstream trend of conducting impervious surface research [10]. Methods for estimating ISA by remote sensing include interpretation classification method, spectral analysis method, and model simulation method [11]. Ridd proposed and constructed the Vegetation-Impervious-Surface-Soil (VIS) model, but did not directly apply the medium-resolution image (10∼100 m) to estimate the impervious surface [12]. With the maturity of spectral mixture analysis (SMA), many scholars have explored the endmember selection and accuracy test [13–15]. So, the linear spectral separation technique for medium-resolution images has been improved, and the VIS-based model was widely applied in urban environments [16–20], urban impervious surface overlay mapping and evaluation [21], and other aspects.

Fuxian Lake is a unique low-latitude and high-altitude plateau lake ecosystem in the world. It is an important international lake to study the mechanism of biodiversity formation. Affected by the East Asian and Southwest monsoon, it is one of the most sensitive representative lakes in response to the global change in the world. Favored by experts and scholars at home and abroad, it has become one of the hot spots of the research on international lakes, and it is also one of the plateau lake basin systems with the research value of the most ecological fragile areas in geosciences of China. However, with the global changes intensified, the level of urbanization increased and the social and economic development accelerated in the basin, the lake body of Fuxian Lake has shrunk, the water level has decreased significantly, and the water area has gradually narrowed in recent years [22]. The land use/land cover changes in the basin are significant, soil erosion and land degradation are serious, and the ecological environment quality of the basin is generally declining [23–25]. The water quality of the entering lake river and lake shore is seriously polluted. Thus, the water quality of the lake is seriously threatened. Therefore, based on these premises, the present study aims at clarifying the relationship between the ISA and water quality and laying a foundation for the improvement and protection of the water quality in the basin. Our goal in this paper was to investigate the historical shifts in ISA in Lake Fuxian between 2006 and 2015. We also examine the spatial scales at which these changes impact water quality by monitoring the water quality data from the local department governing Lake Fuxian throughout the watershed, together with data from four ISA maps (2006–2015). The present study addresses the following research questions: (1) How does the spatial distribution of ISA change in the Fuxian Lake Basin from 2006 to 2015? (2) How do spatial and temporal variations in ISA within and across watersheds influence water quality metrics in the Lake Fuxian watershed? (3) At what spatial scale does the ISA act to influence water quality?

## 2. Materials and Methods

### 2.1. General Situation of the Study Region

**Figure 1:**
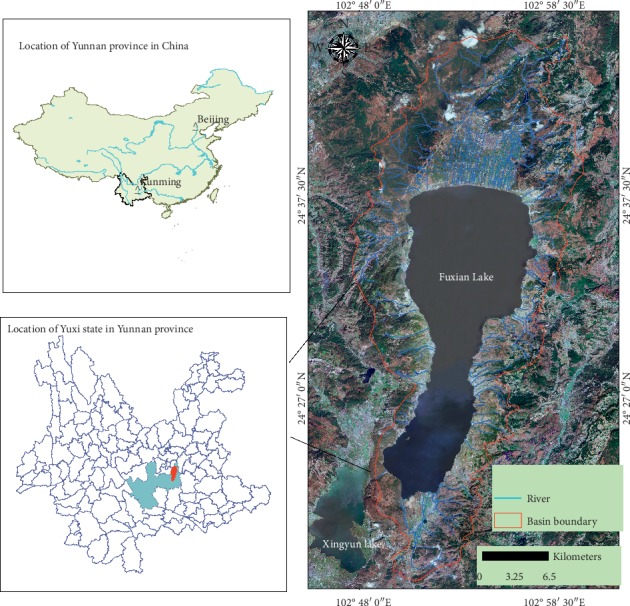
Location of study area.

Located in the center of the Central Yunnan Basin, Yuxi City, central Yunnan Province, Fuxian Lake is China's largest deepwater freshwater lake, the first large lake in the source of the Pearl River, and is the Nanpanjiang River system [22]. Its geographical location is 24°21′28″-24°38′00″N and 102°49′12″-102°57′26″E (Figure 1). As one of the nine plateau lakes in Yunnan Province, Fuxian Lake is the second deepwater lake explored in China and the lake area and water storage amount to the 8th and 3rd in China, respectively [26]. Because of the particularity of its geographical location and its powerful water supply capacity and recreational value, it is known as the “plateau pearl” in central Yunnan, serving as an important resource guarantee for the sustainable social and economic development in central Yunnan, the strategic water resource for the regional development of the Pan-Pearl River Delta, as well as the important strategic source of drinking water in the Pearl River Basin and Southwest China [27]. The Fuxian Lake average water quality meets Class I of the China National Water Quality Standard (CNWQS), i.e., GB3838-2002, and is one of the best natural lakes in China. The vegetation in the basin is mainly secondary vegetation such as grass, shrub, and coniferous forest. The population reaches about 160,300, the rural economy mainly comes from crop production, the main food crops include rice, corn, wheat, and so on, and economic crops include flue-cured tobacco and rapé. The industry is dominated by phosphorus chemical industry, building materials, food processing, and aquatic products, of which phosphorus chemical industry is the pillar industry of this area. The land use type of Fuxian Lake Basin has always been dominated by forests and water areas [22]. However, with the improvement of the urbanization level of the basin, human activities have increased disturbance on the natural environment, such phenomena as reclamation of lakes, deforestation, overexploitation of tourism resources, and the rapid increase of functional buildings result in significant change in land coverage types in Fuxian Lake Basin. ISA has increased dramatically.

### 2.2. Data Sources

By comparing the cloudiness and other noise of the image data of the Landsat-5 TM, Landsat-7 ETM+, and Landsat-8 OLI in the study area, the image acquired by Landsat-7 ETM+ was used for less cloudiness than TM and OLI, which is from http://www.gscloud.cn/. The satellite sensor is Landsat7 ETM+, row 129, column 43. Image acquisition times are as follows: March 8, 2006 (no clouds), February 12, 2009 (cloudiness 0.02%), February 5, 2012 (cloudiness 0.03%), and March 17, 2015 (no clouds).

Seven water quality indicators, such as DO, CODMn, TN, TP, NH3-N, volatile phenol, and petroleum, were selected from the Environmental Protection Bureau of Chengjiang County, Yuxi City. The monitoring points are located at the entrance of Liangwang River, Mayu River, Gehe River, Dongda River, Daicun River, Luju River, Niu Mo River, and Jianshan River into Fuxian Lake. The monitoring frequency is once in the middle of each month.

**Figure 2:**
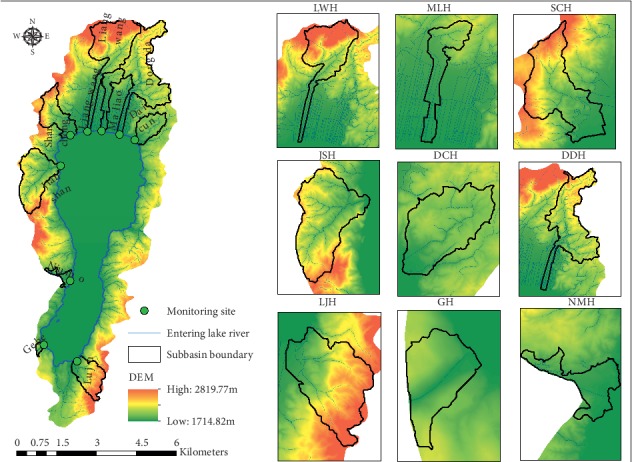
The water quality monitoring site and subbasin location [22].

Based on the topographical features of the basin, subbasin division is performed in the ArcSWAT tool based on the DEM with 2 m grid spacing and water of the 1:10000 Digital Line Graph (DLG) in the study area. The DEM and 1:10000 DLG are provided by Yunnan Provincial Surveying and Mapping Geographic Information Bureau. Considering that the terrain of the north bank of the river basin is flat, in order to ensure that the generated subbasin is in line with the actual situation, water network of 1:10000 DLG should be loaded into the Burn in a stream network to participate in the calculation. The 247 subbasins generated by automatic segmentation are recombined and finally merged into 28, and 9 main river entering the lake are extracted as subbasins (Figure 2). The subbasins include Liangwang River (LWH), Maliao River (MLH), Gehe River (GH), Dongda River (DDH), Daicun River (DCH), Luju River (LJH), Niu Mo River (NMH), and Jianshan River (JSH). The subbasins are used as a subbasin statistical unit for the impervious surface.

The accuracy test data is from the survey results of the geographical survey provided by the Yunnan Provincial Surveying and Mapping Geographic Information Bureau in 2012 and 2015, and the updated land use data are provided by the Yunnan Provincial Department of Land and Resources in 2006 and 2009.

### 2.3. Data Preprocessing

It is also necessary to perform stripe repair, radiation correction, fusion, and masking on the acquired remote sensing image to remove stripping and color distortion noise. *Stripping Noise*. The Landsat-7 ETM+ image data with strip loss is stripped and repaired by the landsat gapfill plug-in based on the ENVI5.1 software platform. The Landsat-7 ETM+ image data is continuous and the visual interpretation effect is good after restoring. Radiation calibration of Landsat data was performed using the Radiometric Calibration tool, and then it was atmospherically calibrated using the FLAASH atmospheric calibration tool. The image fusion method adopts Gram–Schmidt (GS), and Green, Red, and Near Infrared were used for bands composition, and the change of DN value before and after fusion is very small, and the details of the features are clear. If there is high albedo of clouds, sand, or low-reflection water in the study area, it will affect the extraction accuracy of the impervious surface. Therefore, in order to eliminate the interference of water bodies, a modified water body mask was performed using the Modified Normalized Difference Water Index (MNDWI) [28].

The 4-period ISA information was extracted by the Vegetation-Impervious-Surface-Soil (VIS) model. It mainly includes the following steps: minimum noise separation transformation, endmembers selection based on geometric vertices, linear spectral mixture decomposition, and ISA calculation and its accuracy evaluation and analysis. The endmembers can be obtained from the field measurement spectrum library and directly obtained from remote sensing images [29]. Affected by various factors such as the atmosphere, terrain, and sensors, ISA extracted from spectral library has low precision. However, the method of obtaining from the image is simple, of little workload, and of high precision. High albedo, low albedo, vegetation, and soil endmembers types are chosen to perform image Minimum Noise Fraction transformation. And then, the endmembers selection is performed by using three component feature values of about 80% of the original image information.

According to the mathematical definition of the linear mixed model, the three vertex regions of the triangle represent a pure feature type, and the scatter inside the triangle should be a mixed pixel. The MNF images of the first, second, and third components with little correlation and rich information are combined to form a two-dimensional scattergram (Figure 2) and get the image endmember from the scattergram. According to the distribution of different endmember types in the MNF feature space, the sample region where the endmember is located was found, and the mean value of the spectrum of the sample was obtained, and then the surface cover type (endmember) was visually interpreted represented by each triangle vertex region. The linear spectral mixture model (LSMM) was used to decompose the image of the study area by linear spectral mixture model, and the root mean square statistics of high albedo features, low albedo features, vegetation and soil endmember abundance maps, and decomposition results were obtained. The linear spectral mixture model (LSMM) was used to linearly decompose the image of the study area for obtaining the high albedo features, low albedo features, vegetation and soil and abundance maps, and the root mean square statistics of decomposition results. Because the impervious surface in towns is mainly artificial building materials, it includes high-reflection features (such as glass, metal roofs, etc.) and low-reflection features (such as asphalt roads, roofs, and old buildings). Therefore, the impervious surface of the urban area is a linear combination of high albedo components and low albedo components [21]. Therefore, the high and low albedo components are added to obtain an impervious surface:

(1) $$\begin{array}{c} R_{\text{imp},i}=f_{\text{low}}R_{\text{low},i}+f_{\text{high}}R_{\text{high},i}+e_{i}. \end{array}$$

**Figure 3:**
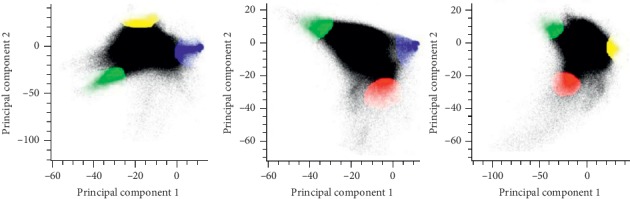
Two-dimensional scattergram.

In the formula, *R*_imp,*i*_ is the spectral reflectance of the *i* band impervious surface; *R*_low,*i*_ and *R*_high,*i*_ are the spectral reflectances of the low albedo and high albedo components in the *i* band, respectively. *f*_low_ and *f*_high_ are the proportions of low albedo and high albedo components; *e*_*i*_ is the residual. According to the above formula and through the band calculation tool, the preliminary impervious surface can be obtained (Figure 3).

The root mean square error (RMS) is used to evaluate the accuracy of the linear spectral mixture model decomposition. The formula for calculating the root mean square error is

(2) $$\begin{array}{c} \text{RMS}={\left[\frac{\sum_{k=1}^{n}{\left(\varepsilon_{i\lambda }\right)}^{2}}{n}\right]}^{1/2}. \end{array}$$

In the formula, RMS is the root mean square of the residual error value and *n* is image bands. The smaller the value of RMS, the smaller the model error. Generally, the average of the root mean square error of the decomposition result must be less than 0.02 [21].

### 2.4. Statistical Analysis Method

The correlation between ISA and water quality is analyzed based on the Pearson correlation analysis method in SPSS software. The water quality indicators (CODMn, TP, TN, and COD) of 9 rivers in the lake basin from 2006 to 2015 were used as response variables, and the proportion of ISA in the basin was interpreted as an explanation variable. The entry probability of the model is 0.05, and the rejection probability is 0.1.

## 3. Results

### 3.1. The Accuracy of the Linear Spectral Mixture Model Decomposition

Experimental statistics show that the RMS maximum of the image decomposition results in 2012 is 0.107, the minimum is 0.003, and the average is 0.017, while the average values for 2006, 2009, and 2015 are 0.016, 0.016, and 0.018, respectively. The values are all less than 0.02, which indicates that the number of endmembers in this study is more suitable, and the spectral values of the endmembers are more accurate, and the decomposition results have practical significance.

According to the rule that pure types of objects are always distributed in the protruding vertex regions of the two-dimensional scatter plot, and then comparing the distribution of triangular vertex regions on the remote sensing image, visually judge the type of surface coverage represented by each triangular vertex region. Each triangle vertex area contains pure pixels. The types of end elements are high albedo, low albedo, vegetation, and soil. The results of selecting end elements are shown in Figure 3 (high albedo is red, low albedo is blue, vegetation is green, and the soil is yellow), and then the three end elements selected by the apex are used for linear spectral decomposition.

### 3.2. Accuracy of the ISA Map

**Figure 4:**
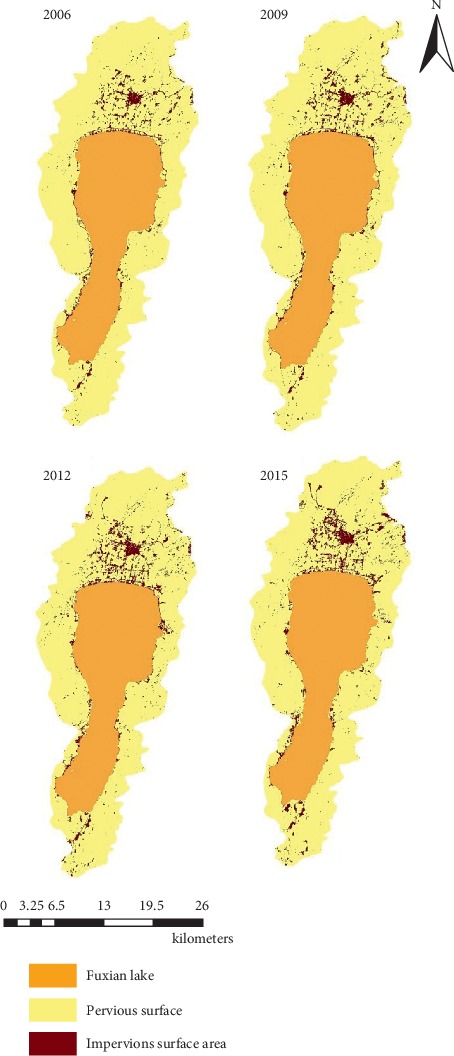
Preliminary impervious surface area.

In order to validate the ISA map accuracy (Figure 4), referring to the current land use map and remote sensing images, the classified data are manually modified to form the final LULC information (Figure 4). The data are from the Yunnan Provincial Bureau of Surveying and Mapping. The land use types are from the results of the first national geographical situation survey in Yunnan Province and surface coverage classification data (water area, desert and bare surface, construction land, arable land, garden land, forest land, structure, grassland, road, and house building (area)), and which is extracted by using QuickBird and the WorldView-2 remote sensing image with resolution of 0.61 and 0.5 meters from 2006 to 2015. Arable land and garden land merged into cropping land (CL); woodland and grassland merge into forestry and grass cover (FGL); buildings (districts), roads, and structures are combined into an impervious surface area (ISA); and the artificially excavated land, desert, and exposed surface are merged into other land (other).

As can be seen from Figure 4, the ISA changed significantly in the the study area from 2006 to 2015, and the area of ISA generally showed an upward trend. The area of ISA was the smallest in 2006 and the largest in 2015. From the perspective of distribution, the ISA is mainly distributed on the north and south shores of Fuxian Lake and gradually expands to the north.

**Figure 5:**
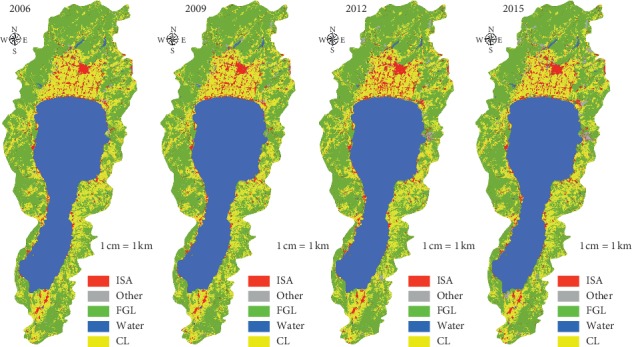
Reference map validation.

As per the spatial overlap analysis on ISA extracted from high-resolution remote sensing images (Figure 5) and ISA extracted from this paper, the overlap area accounts for more than 85% of the total area, which indicate that the accuracy of the ISA extracted in this paper is better.

### 3.3. ISA Quantity Variation Characteristics

**Figure 6:**
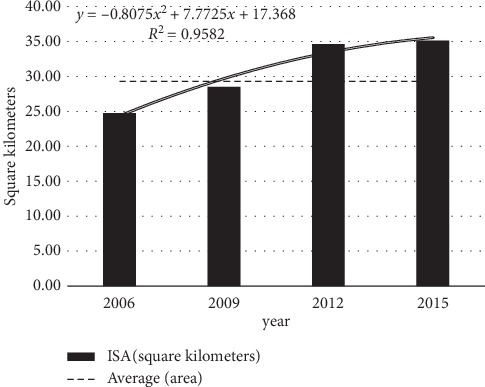
Area changes of ISA in the Fuxian Lake Basin from 2006 to 2015.

**Figure 7:**
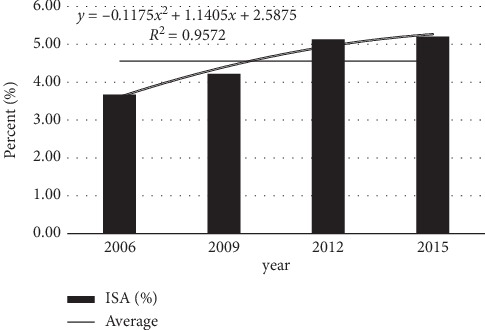
Percent changes of ISA in the Fuxian Lake Basin from 2006 to 2015.

The changes of ISA in the Fuxian Lake Basin in 2006, 2009, 2012, and 2015 are shown in Figures 6 and 7.

It can be seen from Figures 4 and 5 that the ISA generally shows an upward trend from 2006 to 2015. It increased from 24.7 km^2^ in 2006 to 35.1 km^2^ in 2015. From the value anomaly, the ISA in 2006 and 2009 is lower than the multiyear average, and those of the other years are higher than the multiyear average. Impervious surface changes significantly. In 2006, the percentage of ISA accounted for 3.7% of the total area of the basin, while in 2015, it increased rapidly to 5.2%. From 2006 to 2015, the ISA of the Fuxian Lake Basin increased by 1.5%. During the period from 2006 to 2009, the ISA continued to expand, and the area increased from 24.7 km^2^ to 28.5 km^2^. The average annual ISA was 1.3 km^2^, and the average annual growth rate was 5.00%. During the period from 2009 to 2012, the area increased from 28.5 km^2^ to 34.6 km^2^, the average annual growth rate of ISA is 2.0 km^2^, and the average annual growth rate is 7.2%. During the period from 2012 to 2015, the area increased from 34.6 km^2^ to 35.1 km^2^, and the average annual growth of ISA is 0.5 km^2^. The rate was 0.5%. From 2009 to 2012, the ISA increased by a large margin, and the growth level exceeded that of 2006–2009 and 2012–2015.

### 3.4. Spatiotemporal Variation of Water Quality in Subbasins

**Figure 8:**
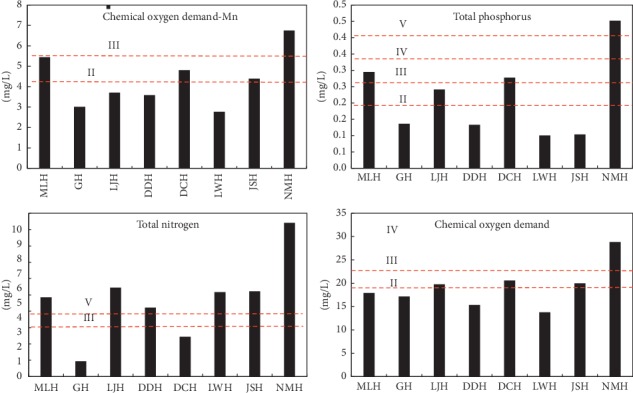
Water quality parameters changes of entering rivers in Fuxian Lake basin.

The water quality parameters changes of the eight subbasins that are mainly located in the lakes in 2015 are shown in Figure 8. The water quality category evaluation of CODMn, TP, TN, and COD in Figure 6 refers to the surface water environmental quality standard (GB 3838-2002).

It can be seen from Figure 8 that the concentration of CODMn in most subbasins is between II and III. Only in Niumohe subbasin, it is higher, reaching level IV. The lowest value appears in the Liangwanghe subbasin. The concentration of TP in each subbasin is in the Class II to the inferior Class V. The Liangwanghe and Jianshanhe subbasins are Class II water quality, the Gehe subbasins and the Dongdahe subbasins are Class III water quality, and the Maliaohe subbasins, the Lujuhezi basin, and the Daicunhe subbasins are Grade IV water quality, and the Niumohe subbasins is inferior to Class V water quality. The concentration of TN is generally higher in the subbasins of Fuxian Lake, and the concentration in the Gehe subbasin is lower, and it is at the level III. The concentration of COD in most subbasins is between II and III. Only the Daicunhe subbasin and the Niumohe subbasin reaches the IV category, and the Niumohe subbasin is the largest.

**Figure 9:**
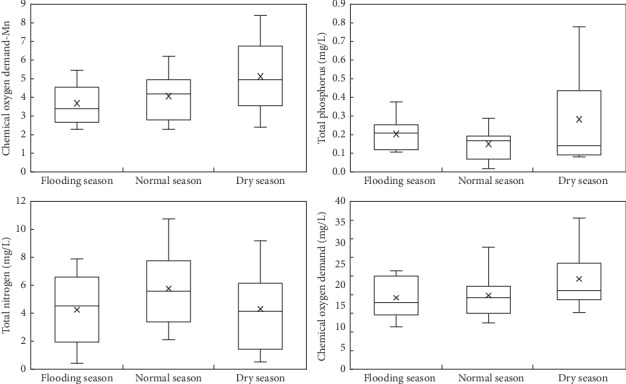
Seasonal change of water quality.

Based on the water quality data of the 12th issue in 2015, the average water quality of the subbasin of Fuxian Lake during the flooding season period (June to September), the normal season period (October to January), and the dry season period (February to May) was further analyzed (Figure 9).

It can be seen from Figure 7 that the average concentration of CODMn and COD is ranked as follows: dry season > normal season > flooding season. The maximum concentration of CODMn appeared in the dry season (8.5 mg/L), and the maximum concentration of COD appeared in the dry season (35.6 mg/L). The average concentration of TP is ranked as follows: dry season > flooding season > normal season. The maximum concentration of TP occurs in the dry season (0.8 mg/L). The average concentration of TN is ranked as follows: normal season > flooding season > dry season. Among the four water quality indicators monitored, the average concentration and maximum concentration of CODMn, TP, and COD were in the dry season. In general, the most serious water pollution in the subbasin of Fuxian Lake is the dry season, followed by the normal season, and the best water quality is during the flooding season.

### 3.5. Correlation between ISA and Water Quality

Correlation analysis was carried out on the water quality of Fuxian Lake and the proportion of ISA by SPSS correlation analysis method. The results are shown in Table 1.

It can be seen from Table 1 that the ISA is significantly positively correlated with permanganate index and TP at 0.05 level, and the correlation coefficients are 0.772 and 0.763, respectively. The correlation is strong, but the correlation with TN and COD is not obvious.

**Figure 10:**
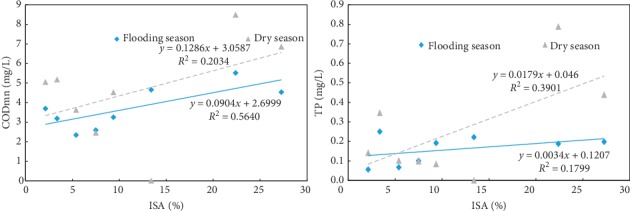
Seasonal correlation between the water quality index concentration and the percentage of ISA.

The water quality indicators monitored in 2015 were classified according to the wet season (June–September) and the dry season (February–May), and the average values of CODMn and TP were obtained in each period. A seasonal correlation analysis was performed between the water quality index concentration and the percentage of ISA in each subbasin. The results are shown in Figure 10.

It can be seen from Figure 8 that the percentage of ISA is positively correlated with the water quality index CODMn and TP, but the seasonal difference is obvious. The linear regression of CODMn, TP, and ISA percentage in the wet season is higher than that in the dry season, indicating that the ISA during the flood season has a good interpretation rate for water quality changes.

**Figure 11:**
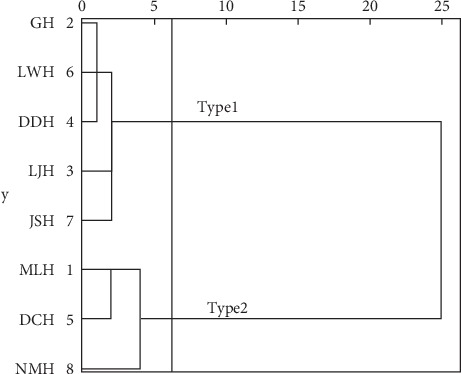
Cluster of percentage of ISA of subbasin and water quality.

**Figure 12:**
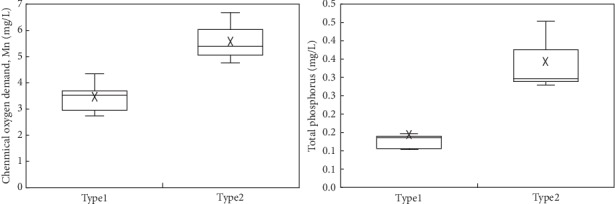
Categories of the ISA ratio and water quality.

In order to analyze the difference and similarity of spatial distribution of water quality indicators in eight subbasins, the percentage of ISA of subbasin and water quality were clustered, and clustering results are shown in Figure 11 and water quality indicators based on clustering result are shown in Figure 12.

It can be seen from Figures 11 and 12 that the relationship between the ISA ratio and water quality of the eight subbasins can be divided into two categories. The first category includes Gehe subbasin, Liangwanghe subbasin, Dongdahe subbasin, Lujuhe subbasin, and Jianshanhe subbasin. The second category includes Maliaohe subbasin, Daicunhe subbasin, and Niumuhe subbasin. The average concentration of water quality indicators in the second subbasin is greater than that in the first subbasin. From the proportion of ISA, the percentage of ISA of Majihe, Daicunhe, and Niumouhe subbasins is 27.3%, 13.4%, and 22.4%, respectively. And in the first subbasin, the percentage of ISA is less than 10% (refer to the results of previous studies [9]). The main city area is at a threshold level of degradation (48%), while the ISA ratio should be reduced from the current 6% to less than 5% in the village farmland area. Therefore, it can be preliminarily believed that the ISA threshold of water quality degradation in the Fuxian Lake Basin is about 10%. When the percentage of the ISA of the subbasin is less than 10%, the water quality of the basin is relatively good. At this time, the river water still has self-purification ability, but when the percentage of the ISA of the subbasin exceeds 10%, the water quality will have a risk of deterioration.

## 4. Discussion

The research on the ISA information extraction and the impact on the water environment on the basin scale provides a new idea and method for urban planning, water environment relief, and watershed management within the basin. How to use high-quality image data and improve existing models to effectively improve the accuracy of the extraction of ISA information will be a work worthy of in-depth discussion and research.

It is a consensus that the ISA has a threshold effect, but the exact threshold has regional differences. Due to the limitation of sampling points, it is necessary to further obtain water quality monitoring data and ISA data of the long-term watershed, which will lay a solid foundation for obtaining the threshold of the ISA of the basin. The method of threshold determination remains to be further studied.

The increase in the level of tempering produces a large number of ISA. This paper only analyzes the response relationship between each water quality index and the ISA. In order to grasp the water environment effect of the ISA, the water environment effect of the ISA in terms of water temperature rise, surface runoff increase, soil erosion, and climate change will be further studied.

Although domestic research has begun to pay attention to the applicability of models to differentiated geographical backgrounds, both model construction and model application need to be explored in the future. On the other hand, compared with foreign countries, domestic research is insufficient to explore the empirical relationship model for long-term monitoring. Due to the short monitoring period and the low frequency of monitoring, it is still difficult to obtain a convincing empirical model. Meanwhile, we should consider the possible effects of the scarce quality of the source in the production of drinking water (i.e., the presence of byproducts due to the disinfection and the high distrust which could grow in the population), by comparing to a wider international panorama [30, 31].

Due to major environmental incidents in China since 2003, such as the chemical explosion in the Songhua River and the cyanobacteria outbreak in Taihu Lake, including some heavy metal pollution issues, China has become an environmental risk society, and measures must be taken; otherwise, water, soil, and gas contamination will cause great problems. From the data of 354 cancer villages surveyed by the Central China Normal University Sustainable Development Research Center, it can be seen that cancer villages are mainly distributed within 3–5 kilometers of the river. Soil and water pollution will cause food security problems and thus pose a threat to human health. Therefore, it is very important to implement water source protection and ecological restoration of the Fuxian Lake Basin for improving the water environment quality of the basin.

## 5. Conclusions

This paper uses the Fuxian Lake watershed as a research area to study the Landsat-7 ETM+ images in 2006, 2009, 2012, and 2015 covering the Fuxian Lake basin. The ISA in the Fuxian Lake watershed was extracted by linear spectral hybrid model (LSMM). The results showed that (1) four endmember types including high albedo, low albedo, vegetation, and soil were selected, and linear spectra were used. The linear spectral mixture model (LSMM) extracts the ISA in the Fuxian Lake basin with an average RMS of less than 0.02. It is indicated that the number of end elements in this study is more suitable, and the spectral value of end elements is more accurate. The model can effectively extract the ISA of the Fuxian Lake watershed.

From 2006 to 2015, the ISA of the study area showed an overall upward trend. It increased from 3.7% in 2006 to 5.2% in 2015, an increase of 1.5%. With the improvement of the urbanization level of the river basin, the implementation of a large number of land development projects such as real estate, public facilities, and roads in the urban area has occupied a certain amount of planted land and forest and grass cover, and there have been a large number of hardened surface and differences in subbasin changes obviously. The percentage of ISA in the Maliaohe subbasin and Niumohe subbasin exceeded 20%, which were 27.3% and 22.4%, respectively.

The concentrated distribution of phosphate deposits in the Dongdahe and Daicun River basins in the phosphate mining area makes the artificially burrowed land, bare surface, and ISA closely related to TP, which becomes an important factor affecting TP. Cropping land, forest cover, and ISA in village farmland have significant effects on TP and TN. The reduction of cropping land has not led to better water quality, indicating that one of the important reasons for the deterioration of water quality in this area may be the rapid increase of ISA. The forest and grass cover can play a good role in reducing water quality. The increase of other land types will also worsen the water quality, but the sensitivity between the two is low.

The water quality of the Fuxian Lake subbasin has obvious spatial and temporal heterogeneity. In terms of space, the concentration of various water quality indicators in the Niumohe subbasin and Maliaohe subbasin with high level of urbanization is relatively high. In terms of time, the CODMn and TP in the Fuxian Lake subbasin are as follows. The COD has the highest concentration in the dry season, the second in the normal season, and the lowest in the flooding season. Extreme values of TP are found in almost all subbasins, especially in Type 2 subbasins. The presence of ISA does not justify the high value of the TP parameter but could explain the COD due to leaching of road contaminants and highways such as oils, fuels, and others.

The percentage of ISA in each subbasin was significantly positively correlated with water quality index (permanganate index, TP), and the correlation between the wet season was higher than that of the dry season. The results of cluster analysis of the percentage of ISA and water quality in each subbasin indicate that the ISA threshold of water quality deterioration in Fuxian Lake Basin is about 10%.

## Figures and Tables

**Table 1:** Relationship between water quality indicators and percentage of ISA.

Indicators	CODMn	TP	TN	COD
Correlation coefficient	0.772^*∗*^	0.763^*∗*^	0.328	0.428
Significance	0.025	0.028	0.428	0.290

^*∗*^Significant correlation at the 0.05 level (both sides).

## Data Availability

The water quality (.XLS), ISA data (.XLS), and remote-sensing image (.img) data used to support the findings of this study are currently under embargo, while the research findings are commercialized. Requests for data, 1 month after publication of this article, will be considered by the corresponding author.
